# *Anisakis simplex* (sensu lato) and *Hysterothylacium cornutum* (Nematoda: Ascaridoidea) in adult Atlantic bluefin tuna (*Thunnus thynnus*) caught in Norway

**DOI:** 10.1016/j.fawpar.2025.e00261

**Published:** 2025-04-14

**Authors:** Miguel Bao, Arne Levsen, Lucilla Giulietti, Martin Wiech, Keno Ferter, Egil Karlsbakk, Paolo Cipriani

**Affiliations:** a Institute of Marine Research (IMR), PO Box 1870 Nordnes, N-5817 Bergen, Norway; b Department of Biological Sciences, University of Bergen, Bergen, Norway; c Sapienza University of Rome, Rome, Italy

**Keywords:** *Anisakis*, *Hysterothylacium*, *Thunnus thynnus*, Bluefin tuna, Food safety, Pathology

## Abstract

The Atlantic bluefin tuna *Thunnus thynnus* is one of the largest and most valuable fish species in the Atlantic Ocean. Its meat is highly appreciated worldwide, particularly in Japan, where it is commonly consumed raw as sushi or sashimi. Here, we investigated the occurrence and species composition of parasitic nematodes in the viscera of adult Atlantic bluefin tuna caught off western Norway. The zoonotic nematodes *Anisakis simplex* (sensu stricto) and *Anisakis pegreffii* are reported for the first time in wild large adult specimens. Findings suggest that both anisakids appear unable to penetrate the stomach wall of large tuna. Instead, they remain attached and are associated with pathologies, including crater-like ulcers and tumours, sometimes filled with cyst-like decomposition products. A few anisakid larvae were, however, found encapsulated on the intestine and caeca, suggesting that they may have penetrated the thinner walls of the digestive tract there. These results highlight the need for further research on tuna's muscle to rule out any food safety concerns. Additionally, the raphidascaridid nematode *Hysterothylacium cornutum* and a single 4th-stage larva of *H. aduncum*, were identified in the tuna stomachs. Partial LSU rDNA, mtDNA *cox*2 and ITS rDNA sequences of *H. cornutum* are reported for the first time. These sequences may aid resolving the taxonomy of the genus *Hysterothylacium* and unravelling the parasite's life cycle in future studies.

## Highlights

• *A. simplex* (sensu stricto) and *A. pegreffii* are first reported in large adult bluefin tuna.
• *A. simplex* (sensu lato) appears unable to penetrate the stomach wall but may cause crater-like ulcers there.
• Few larvae on caeca and intestine wall suggest ability to trespass the intestine wall.
• Further research on tuna's muscle is recommended to rule out any food safety concern.
• First molecular characterization of the tuna worm *Hysterothylacium cornutum*.

## 1 Introduction

The Atlantic bluefin tuna (*Thunnus thynnus*) is a large, highly migratory species with high economic importance (Fromentin and Powers, 2005). Individuals can grow to over 3 m in length and have a maximum theoretical life span of approximately 40–50 years (Santamaria et al., 2009). In North Atlantic, there are two major, genetically distinct stocks, i. e. the Eastern stock that spawns in the Mediterranean Sea and the Western stock that spawns in the Gulf of Mexico (Block et al., 2005). Norway has a long history of fishing for Atlantic bluefin tuna with catches up to 15,000 tons per year in the middle of the last century (Nøttestad et al., 2017), but following significant stock declines, bluefin tuna almost disappeared from Nordic waters. However, during the last 15 years, increasing numbers of bluefin tuna are observed and caught in Nordic waters (Nøttestad et al., 2020), and the species has once again the potential to be an important fishery resource in Norway. Commercial fisheries are mainly conducted using purse seine and rod-and-reel, alongside recreational fishing (Sistiaga et al., 2025). Atlantic bluefin tuna, a premium ingredient in sushi and sashimi, is consumed domestically in Norway and predominantly exported to international raw fish markets (Sistiaga et al., 2025). Typically, the fish is ice-stored and sold fresh. Recently, Norway has focused on developing live storage fisheries from purse seine catches to enhance product quality and ensure on-demand supply (Sistiaga et al., 2025). Consequently, because fresh bluefin tuna is preferably consumed raw, there arise food safety concerns related to fish-borne zoonotic parasites, such as anisakid nematodes.

The third larval stage of the zoonotic nematode species *Anisakis simplex* (s. s.) and *A. pegreffii* (Nematoda: Ascaridoidea: Anisakidae) commonly occurs in many commercially harvested fish species in North Atlantic waters (Mattiucci et al., 2018). Generally, small planktonic or semiplanktonic crustaceans act as intermediate hosts, fish and cephalopods as paratenic hosts, and cetaceans as definitive hosts in the parasite's life cycle (Mattiucci et al., 2018). Humans may become accidental hosts through consumption of raw or undercooked seafood products containing viable *Anisakis* larvae (Kołodziejczyk et al., 2020). However, properly heating or freezing seafood guarantee the inactivation of *Anisakis* spp. larvae and are considered the most effective prevention methods (EFSA BIOHAZ Panel et al., 2024).

Despite *A. simplex* (s. s.) and *A. pegreffii* being reported in bluefin tuna (Mattiucci et al., 1997; Mladineo et al., 2011; Mladineo and Poljak, 2014), no research has been conducted so far on their occurrence in large adult commercial specimens. In addition, while the parasites' ability to cause gastrointestinal and allergic disorders in humans is well studied and documented (Bao et al., 2019), their role as possible pathogens or immune activation agents in fish has received comparatively little attention (Buchmann and Mehrdana, 2016).

Other common parasitic nematodes in North Atlantic fishes belong to genus *Hysterothylacium* (Nematoda: Ascaridoidea: Raphidascarididae), particularly the species *H. aduncum* (Klimpel and Rückert, 2005). These nematodes use fishes both as paratenic and definitive hosts and are considered non-zoonotic (Cavallero et al., 2020).

The present study aimed to map the occurrence and infection site of ascaridoid nematodes in the viscera of a large, highly valuable, and rarely studied host, the Atlantic bluefin tuna.

## 2 Materials and methods

### 2.1 Sampling

Atlantic bluefin tunas (*N* = 11) were caught by local fishermen off western Norway during August – October 2022 and 2023. Total length (mean ± SD (range) in cm) of fishes was 251 ± 11 (240–270). The total weight (mean ± SD (range) in kg) of six tunas was 256 ± 33 (210–292). The stomach (N = 11), intestine (*N* = 8), caeca (*N* = 5) and liver (*N* = 4) were examined for the occurrence of *Anisakis* spp. larvae. Specifically, the stomach was opened, food contents were carefully removed, and visually inspected, with only those larvae attached to the wall considered. Visceral organs, i. e. intestines, caeca and liver, were inspected for anisakids by candling and UV-press detection methods (ISO 23036-1:2021) (ISO, 2021; Levsen et al., 2005). In brief, each organ was placed in a separate transparent plastic bag and pressed to a thin layer with a hydraulic press, then frozen at −20 °C for over 24 h to kill of any present parasite. After thawing, samples were first visually checked for parasites on a light table (candling), and then visually examined for anisakid larvae using a UV light device in a dark room. The UV-press method is an international standard that uses the fluorescence of frozen and inactivated anisakids for its quantitative detection in fishery products. Additionally, these organs were visually inspected for any signs of pathology associated with parasite presence.

In addition, the occurrence of *Hysterothylacium* sp. raphidascaridids free in the stomach and intestinal lumen was also visually inspected under bright light conditions. Prevalence and mean abundance of ascaridoid infections were calculated following Bush et al., 1997.

### 2.2 Identification

*Anisakis* and *Hysterothylacium* larvae were morphologically assigned to larval type following Berland (1961), using morphological characters such as presence or absence of boring tooth/lips, intestinal caecum, ventricle, ventricular appendix, cuticle ornamentation and terminal mucron/“cactus” tail, as well as the position of the excretory pore relative to the boring tooth/lip. The *Hysterothylacium* specimens were morphologically assigned to species according to main diagnostic characters such as shape and ornamentation of lips, shape and length of intestinal caecum, ventriculus and ventricular appendix, shape of the tail, and cuticle ornamentation (Baylis, 1923; Berland, 1961; Deardorff and Overstreet, 1981).

DNA was extracted from *Anisakis* larvae (*N* = 37) and adult specimens of *Hysterothylacium* cf. *cornutum* (*N* = 4) using the DNeasy Blood & Tissue Kit (QIAGEN GmbH, Hilden, Germany) following manufacturer's instructions. The mtDNA *cox*2 sequences of the *Anisakis* larvae and one *Hysterothylacium* specimen were amplified using the primers 211F (5’-TTTTCTAGTTATATAGATTGRTTTYAT-3′) and 210R (5′-CACCAACTCTTAAAATTATC-3′) (Nadler and Hudspeth, 2000), following procedures of Bao et al., 2022.

For the *Hysterothylacium* specimens, the entire internal transcribed spacer (ITS) rDNA of all four specimens and the partial large subunit (LSU) rDNA of one specimen were amplified. The ITS rDNA was amplified using the primers NC5F (5′ – GTAGGTGAACCTGCGGAAGGATCATT-3′) and NC2R (5’-TTAGTTTCTTTTCCTCCGCT −3′) (Zhu et al., 2000), while the LSU rDNA was amplified using the primers 28SF (5’-AGCGGAGGAAAAGAAACTAA-3′) and 28SR (5’-ATCCGTGTTTCAAGACGGG-3′) (Nadler and Hudspeth, 1998), following Bao et al., 2022 and Li et al., 2018. PCR products were sent for purification and sequencing, using the primers 210R, NC5F, NC2R and 28SF, to Eurofins (Cologne, Germany). Sequences were searched for similarity by BLAST (Basic Local Alignment Search Tool) of the NCBI (National Center for Biotechnology Information).

### 2.3 Phylogenetic analyses

The combined ITS-1 and ITS-2 sequence data were manually obtained by trimming the ITS sequence of the *Hysterothylacium* cf. *cornutum* specimen. The sequence was aligned with selected sequences from GenBank using CLUSTAL W in MEGA 11.0.10 (Tamura et al., 2021) (Table 1). High similarity scores in the BLAST were employed to select the sequences, taking into consideration the findings of previous studies (Palomba et al., 2021; Shamsi, 2017). Phylogenetic analysis was performed by Bayesian Markov Chain Monte Carlo in BEAST v1.10.4 (see tutorial at https://beast.community/index.html) (Suchard et al., 2018). The best-fit substitution model was selected using the Bayesian Information Criterion as implemented in MEGA 11.0.10. The BEAST input files were previously generated in BEAUti by introducing the best substitution models available, i. e. HKY + G, Yule speciation process as tree prior, and otherwise default settings. Effective sample sizes of parameters (cut-off values >200) were checked in Tracer v.1.7.2. The maximum clade credibility trees were constructed in TreeAnnotator v1.10.4, and Figtree v1.4.4 was used to visualize them. Phylogenetic analyses of the LSU rDNA and mtDNA *cox*2 datasets were performed as described above.

**Table 1:** Nematode specimens used for the Bayesian inference analysis of the combined ITS-1 and ITS-2 regions.

Parasite	Stage	Host	Locality	GenBank accession number	Reference
*Hysterothylacium cornutum*	Adult	*Thunnus thynnus*	Western Norway	PV235304	This study
*Hysterothylacium australe*	Adult	*Seriola lalandi*	South Australia	HE862216-HE862225	Shamsi, 2017
*Hysterothylacium persicum*	–	*Scomber australasicus*	Australia	MW315521	Unpublished
*Hysterothylacium* sp.	–	*Coryphaena hippurus*	South Carolina	MF668897	Unpublished
*Hysterothylacium* sp. type X	L3	*Priacanthus arenatus*	Brazil	KU594489	Pantoja et al., 2016
*Hysterothylacium* sp. type XV	L3	*Elops saurus*	Brazil	KU594491	Pantoja et al., 2016
*Hysterothylacium amoyense*	L3	*Halieutaea stellata*	South China Sea	KP252131	Li et al., 2016

## 3 Results

### 3.1 Identification

*Anisakis* larvae (*N* > 100) were morphologically assigned to *Anisakis* type I larva sensu Berland, 1961 (i. e. *A. simplex* sensu lato (s. l.)). Out of the 37 larvae genetically identified, 36 were identified as *A. simplex* (s. s.) and one larva as *A. pegreffii* (i. e. the mtDNA *cox*2 sequences obtained (562 bp) showed 98.93–100 % identity with *A. simplex* (s. s.) (e. g. accession number MN961172) and one sequence showed 100 % identity with *A. pegreffii* (e. g. accession number KR149283) deposited in GenBank). The sequences were deposited in GenBank under accession numbers: PV243631- PV243657.

**Fig. 1:**
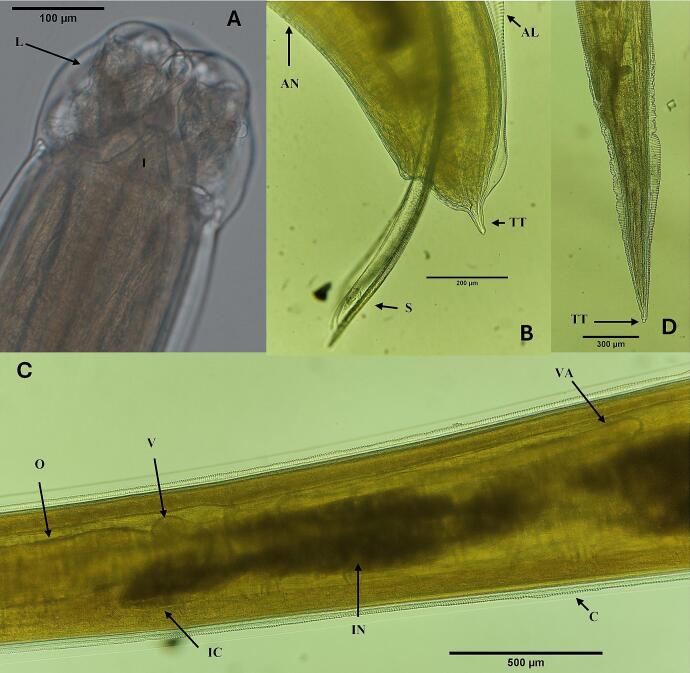
Adult male of *Hysterothylacium cornutum* (isolate TTHC2) from Atlantic bluefin tuna. A: cephalic end from ventral view showing interlabia and subventral lips. B: caudal region, showing characteristic short and conical tail ending in a conical appendage at the tip, spicules, alae and ventral annulations (black arrow). C: shape and arrangement of ventriculus region with diverticula. D: caudal region of an adult female of *H. cornutum* (isolate TTHC4), showing characteristic long and slender tail ending to a conical point at the tip. Abbreviations: AL, alae, AN, annulations, C, cuticle, I, interlabia; IC, intestinal caecum, IN, intestine, L, lip; V, ventriculus, VA, ventricular appendix, O, oesophagous, S, spicule, TT, tail tip.

A total of four adult nematodes belonging to genus *Hysterothylacium* were morphologically identified as *H. cornutum* (Stossich, 1904) Deardorff and Overstreet, 1981 based on key diagnostic features: i) a dorsal lip with two anterior lobules; ii) a short intestinal caecum, and ventriculus with long ventricular appendix; iii) presence of a short, conical tail in males, which narrows abruptly behind the anus with a conical projection at the tip, while in females, the tail is long and slender, tapering gradually from the anus to a conical point; and iv) a serrated cuticle resembling minute saw-teeth (Fig. 1). The worms represented two males (48–49 mm long) and two females (40–47 mm long). Additionally, one nematode was morphologically identified as *Hysterothylacium aduncum* (Rudolphi, 1802) stage IV. No genetic identification was performed on that specimen.

Partial LSU, *cox*2 and ITS sequences of *H. cornutum* were deposited in GenBank under the following accession numbers: LSU: PV239777; *cox*2: PV243630; ITS: PV235304 - PV235305. The ITS rDNA sequences of the four *H. cornutum* specimens were 100 % identical. The partial LSU sequence (702 bp) showed 98.6 % identity with *Hysterothylacium pelagicum* from the common dolphinfish *Coryphaena hippurus* from the Gulf Coast of Mississippi (United States) (accession number U94761). The ITS sequences (863–1014 bp) showed 99.0 % identity to *Hysterothylacium* sp. from *C. hippurus* from South Carolina (United States) (accession number MF668897). The *cox*2 sequence (556 bp) did not show significant genetic similarity in the BLAST analysis (i.e., less than 83 % identity to *Hysterothylacium deardorffoverstreetorum*, accession number KU886687).

### 3.2 Ascaridoid infection characteristics

**Fig. 2:**
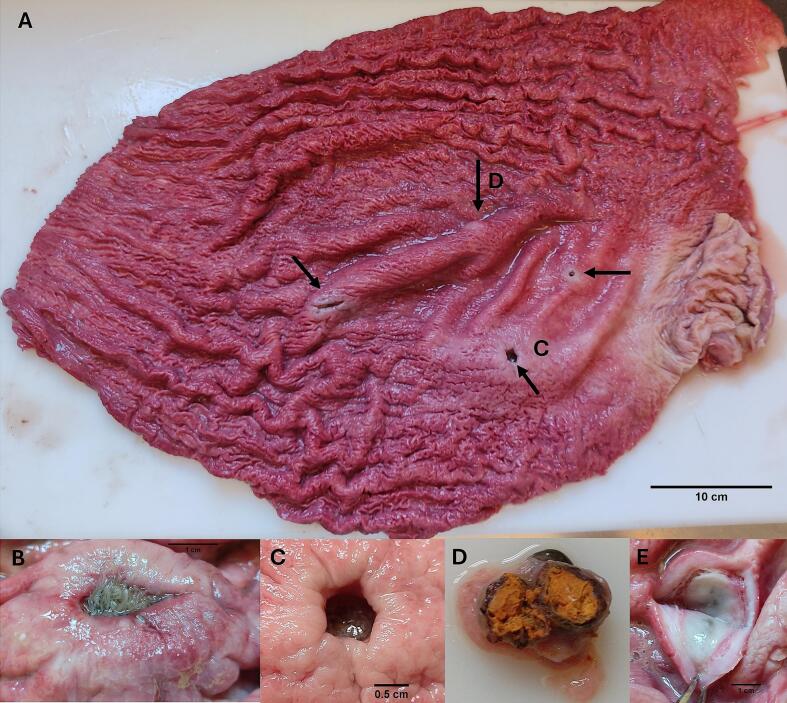
A: Crater-like ulcers (black arrows) caused by *A. simplex* (*s. l.*) within the stomach wall of Atlantic bluefin tuna. B: Living *A. simplex* (*s. l.*) larvae clustered within a crater-like ulcer. C: Crater-like ulcer, with living larvae inside the hole. D: Solid cyst containing larval decomposition products within a tumour-like lesion. E: Tumour-like lesion, apparently resulting from ulcer healing processes.

One to four crater-like ulcers in the stomach wall were observed in 5 out the 11 (45 %) tunas, with an average of 2.4 (Fig. 2A). In most of these ulcers, clusters of living third-stage *A. simplex* (s. l.) larvae occurred, with their head-ends deeply embedded in the stomach and the tails pointing to the lumen (Fig. 2B, C). Old apparently healing lesions were also observed (Fig. 2D, E). Some of the ulcerative lesions emitted a putrid smell. In other organs, three and two *A. simplex* (s. l.) larvae were found encapsulated on the intestine (prevalence (P) = 13 %) and caeca (*P* = 20 %), respectively, of a single tuna which also had the stomach infected (Table 2). No liver was found infected with anisakid larvae. Several (*N* > 20) *Anisakis* (s. l.) larvae were also observed free within the stomach and intestine lumens, typically associated with ingested prey, and therefore not counted.

**Table 2:** Infection descriptors of *Anisakis simplex* (s. l.) and *Hysterothylacium cornutum* in Atlantic bluefin tuna from off Western Norway per infection site.

Parasite species	Infection site	Prevalence	Mean abundance ± SD (range)
*A. simplex* (*s. l.*)	Stomach wall	36 % (4 out of 11)	NA
	Intestine	13 % (1 out of 8)	0.4 ± 1.1 (0–3)
	Caeca	20 % (1 out of 5)	0.3 ± 0.8 (0–2)
	Liver	0 % (0 out of 4)	0
*H. cornutum*	Stomach	18 % (2 out of 11)	0.4 ± 0.9 (0–3)
	Intestine	0 % (0 out of 8)	0

NA = not available. Larvae clustered within the crater-like ulcers were not counted.

*H. cornutum* specimens were found free in the stomach lumen of two tunas (prevalence = 18 %), causing apparently no pathology to the fish (Table 2). No *H. cornutum* was found in the intestine. The fourth-stage *H. aduncum* larva was found free in the stomach lumen.

### 3.3 Phylogenetic analyses

**Fig. 3:**
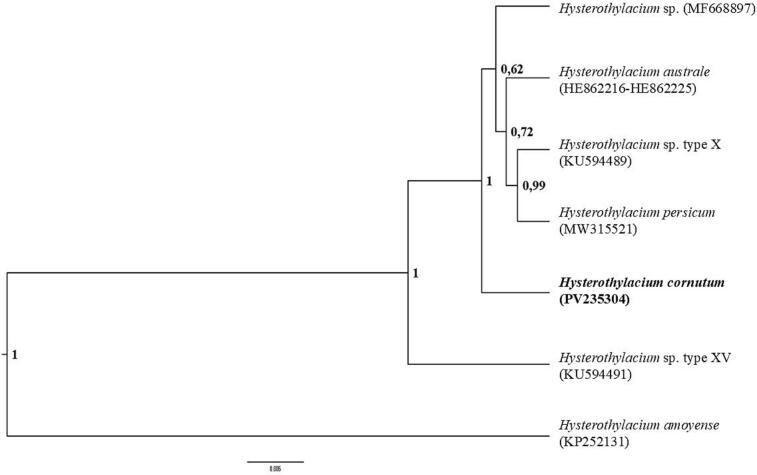
Phylogenetic tree inferred by bayesian inference based on the combined ITS-1 and ITS-2 sequences of *H. cornutum*, with respect to selected sequences retrieved from the GenBank. Nodal support is indicated as posterior probabilities. Branch length scale indicates number of substitutions per site.

The bayesian inference (BI) phylogenetic tree based on the combined ITS-1 and ITS-2 region showed *H. cornutum* to be closest to a clade containing *Hysterothylacium* sp. identified from *C. hippurus*, *Hysterothylacium australe* identified from the yellowtail amberjack *Seriola lalandi*, *Hysterothylacium* sp. type X identified from the Atlantic bigeye *Priacanthus arenatus* and *Hysterothylacium persicum* identified from the blue mackerel *Scomber australasicus* (Fig. 3). A tree based on a partial LSU rDNA region yielded limited information, aside from confirming the close relationship between *H. cornutum* and *H. pelagicum* identified from *C. hippurus* (accession number MF668810). The BI tree of the mtDNA *cox*2 dataset showed poorly supported nodes. Both trees are therefore not presented.

## 4 Discussion

To the best of our knowledge, this study presents the first epidemiological data of *A. simplex* (s. s.) and *A. pegreffii* in wild large adult Atlantic bluefin tuna. Using the UV-press method (ISO, 2021), we reported *A. simplex* (s. l.) infections in the stomach (*P* = 36 %), and visceral cavity around the intestines (*P* = 13 %) as well as the caeca (P = 20 %). Previous studies focused only on juvenile bluefin tuna and employed less efficient gross visual examination for anisakid detection. Wild juvenile bluefin tuna caught in the Adriatic Sea and transferred to sea-cages for fattening, carried *A. simplex* (s. l.) in their viscera, specifically around the intestines, at prevalences (P) ranging from 17 % to 33 % (Mladineo et al., 2011; Mladineo and Poljak, 2014). Similarly, the stomach of wild juvenile bluefin tuna caught off Brazil was found infected with *Anisakis* spp. (*P* = 12 %), whilst other visceral organs such as the intestine, liver and caecum harboured no larvae (Knoff et al., 2017). The present study represents the first documentation of *A. simplex* (s. s.) infecting the caeca of bluefin tuna. The species *A. pegreffii*, *A. simplex* (s. s.) and *A. typica* have been genetically identified from bluefin tuna caught or fattened in cages in the Mediterranean Sea (Farjallah et al., 2008; Mattiucci et al., 1997; Mladineo and Poljak, 2014; Vardić Smrzlić et al., 2012), as well as *A. pegreffii* and *A. typica* in wild juvenile bluefin tuna caught off Brazil (Mattiucci et al., 2002). Thus, the present study extends the occurrence range of *A. simplex* (s. s.) and *A. pegreffii* to also include large adult bluefin tuna fished off western Norway.

Our observations suggest that crater-like ulcers in the stomachs of bluefin tuna are caused by *Anisakis* larvae, as they were found clustered within these lesions. *Anisakis simplex* (s. l.) larvae digested free from prey penetrate the gut wall of fish paratenic hosts, and the stomach is a major site of penetration (Bao et al., 2015; Levsen and Berland, 2012). Similar “craters” associated with *A. simplex* (s. l.) infections have also been observed in large cod *Gadus morhua* in Norway (Berland, 1981); a condition now commonly referred to as the “stomach crater syndrome” (Levsen and Berland, 2012). It seems possible that in large (old) hosts, such as large tuna and cod, the *A. simplex* (s. l.) larvae are not able to penetrate the thick stomach wall, instead producing the craters of this syndrome (see also Berland, 2006). This phenomenon has not been previously documented in tuna. Similarly, ulcers caused by *Anisakis* spp. have been observed in the proventriculus of fish eating birds (e. g. cormorants (EK unpublished)), stomach of seals (Smith, 1999) and are frequently observed in their cetacean definitive hosts (Pons-Bordas et al., 2020; Ryeng et al., 2022; Smith, 1999,). Remarkably, *Anisakis* larvae accumulate in craters rather than dispersing randomly in the stomach wall, which indicates an aggregating behaviour. Interestingly, clusters forming masses of hundreds of aggregating larvae in the viscera of several fish species such as European hake (*Merluccius merluccius*) or allis shad (*Alosa alosa*) have also been documented (Bao et al., 2015; Cipriani et al., 2025). The mechanisms driving this parasite aggregation behaviour are unknown.

Tissue damage and a putrid smell associated with the ulcers in bluefin tuna suggest necroses and hence pathology. It is unknown how this condition impacts the fish, but the presence of some apparently old, healing ulcers suggests that bluefin tuna eventually may kill and reabsorb the *Anisakis simplex* (s. l.) larvae. Indeed, Mladineo et al. (2011) found that the prevalence of *Anisakis* sp. declined from 33 % to 17 % in wild-caught juvenile tuna that were kept in sea cages for 1.5–2 years. The finding strongly suggests that a significant number of larvae died during the period.

Bluefin tuna trophically acquires *Anisakis* larvae through infected prey. In the Mediterranean, common prey such as the carangid *Trachurus* spp. or the ommastrephid squid *Illex coindetti* (Battaglia et al., 2013; Karakulak et al., 2009; Sorell et al., 2017), are known hosts of *A. pegreffii* larvae (see Table 2 of Mattiucci et al., 2018 and references therein). In Norwegian waters, Atlantic mackerel *Scomber scombrus* and Atlantic herring (*Clupea harengus*) seem to be important prey species (Nøttestad et al., 2017, Nøttestad et al., 2020), and are well-documented hosts of *A. simplex* (s. s.) (Levsen et al., 2018a, Levsen et al., 2018b). Considering the large estimated daily food intake of bluefin tuna (1–2.4 Kg/day) (Sorell et al., 2017; Turcotte et al., 2023), the observed low infection rates of *A. simplex* (s. l.) are unexpected. This suggests that large bluefin tuna are possibly dead-end hosts in the life cycle of *Anisakis* nematodes, as has been suggested for other large-sized top predator fish such as the swordfish *Xiphias gladius* (Mattiucci et al., 2014). Although bluefin tuna continuously acquire large numbers of *Anisakis* spp. larvae by preying on infected fish and squid, the vast majority of these larvae become trapped in the stomach wall, remain transient, or are eliminated. Moreover, large bluefin tuna are rarely predated by marine mammals (Guinet et al., 2007).

Atlantic bluefin tuna annual migration in the NE Atlantic has recently been studied by satellite-tagging (Ferter et al., 2024). Data showed that they cross the Strait of Gibraltar and enter the Mediterranean Sea in May, remaining there for 1–2 months before leaving again for their northward migration to Norwegian waters, which they reach in late summer/early autumn, on average, in 40 days (Ferter et al., 2024).

*Anisakis pegreffii* is the dominant anisakid species in the Mediterranean Sea and co-occurs with *A. simplex* (s. s.) in hosts from western Iberia (Mattiucci et al., 2018; Ramilo et al., 2023). In contrast, *A. simplex* (s. s.) becomes the dominant species in northern NE Atlantic waters (Levsen et al., 2020). In the present study, most of the larvae appeared to be *A. simplex* (s. s.), with only a single larva identified as *A. pegreffii*. Most likely, the *A. pegreffii* larva was acquired in the Mediterranean Sea or western Iberia waters through predation on infected prey (fish, cephalopods or crustacean hosts). However, its anecdotal occurrence suggests that most *A. pegreffii* larvae ingested during the Mediterranean/Iberian phase are expelled or killed during the 1 to 2 months it takes for the tuna to reach Norway (Ferter et al., 2024). This finding is significant, as it suggests that *Anisakis* larvae may not encapsulate or bioaccumulate in bluefin tuna over extended periods. Consequently, the use of anisakids as indirect biological indicators for bluefin tuna stock migration routes (Mackenzie and Hemmingsen, 2015) could be limited. This also implies that any *Anisakis* larvae potentially reaching the muscle during the juvenile phase would likely be dead by the time the tuna reaches adulthood.

The presence of *Anisakis* larvae encapsulated on the caeca demonstrates that some can penetrate the digestive tract, likely in the intestine, where additional larvae were found both in the contents and attached to the wall. Therefore, it cannot be entirely ruled out that some *Anisakis* larvae may reach the muscle, posing a health risk to sushi and sashimi consumers. While *Anisakis* larvae have been documented in the muscle of related scombrids such as Atlantic mackerel and skipjack tuna *Katsuwonus pelamis* (Levsen et al., 2018a; Takano et al., 2024), there is currently no data available regarding their presence in the muscle of adult bluefin tuna. Neither are there any cases of anisakiasis implicating bluefin tuna preparations. Bluefin tuna is a large and highly valued fish, making it impractical and unaffordable to examine the entire musculature for *Anisakis* larvae. Consequently, only the stomachs of 11 specimens, along with the intestines (*N* = 8), caeca (*N* = 5) and liver (*N* = 4) of some specimens, were opportunistically studied, representing a limitation of the present study. Further research including larger sample sizes is recommended to improve the informative impact of the findings. Examination of the internal organs and as well as the hypaxial (largely comprising the belly flaps) and perianal muscles should be prioritized, since the distance for the larvae to pass, is shortest between the viscera and these muscle parts. *Anisakis simplex* (s. s.) accumulating around the vent has been documented in fish species such as Atlantic salmon *Salmo salar* (Noguera et al., 2009).

*Hysterothylacium cornutum* was described by Stossich (1904) as *Ascaris cornuta* from the stomach of a bluefin tuna caught in the Adriatic Sea. Its description was further detailed by Baylis (1923), who transferred the species to the genus *Contracaecum* as *C. cornutum*. Dollfus (1933) erected the genus *Thynnascaris* for *T. legendrei*, a species described from the stomach of the albacore *Thunnus alalunga* in the Bay of Biscay, assuming that it lacked a ventricular appendix. However, in 1935, Dollfus recognized that *T. legendrei* did possess a ventricular appendix and subsequently reclassified it under *Contracaecum*, retaining *Thynnascaris* as a subgenus (Dollfus, 1935). Unaware of Baylis's earlier description of *C. cornutum*, Dollfus maintained *C. legendrei*, which was later synonymized with *C. cornutum* by Berland (1961). The species was finally transferred to the genus *Hysterothylacium* by Deardorff and Overstreet, 1981.

*Hysterothylacium cornutum* appears specific to the genus *Thunnus* (Bruce and Cannon, 1989). It has been reported worldwide from the bluefin tuna (type host), albacore, yellowfin tuna *Thunnus albacares*, and the southern bluefin tuna *Thunnus maccoyii*, in diverse locations such as the Adriatic, Bay of Biscay, eastern North Atlantic, North and Baltic Seas, North Pacific, Hawaii, off eastern Australia and from the eastern North Pacific (Bruce and Cannon, 1989; Deardorff and Overstreet, 1982; Moravec and Nagasawa, 2000). In 1955, Berland reported a 67 % prevalence of *H. cornutum* in bluefin tuna from western Norway (Berland, 1961), higher than the 18 % observed in the present study. Adult *Hysterothylacium* individuals reproduce and subsequently die, rapidly disappearing from the fish (Bao et al., 2021). Thus, the differences in prevalence observed by Berland and in this study may be related to the timing of the parasite infection and other unknown ecological factors (i. e. the life cycle of *H. cornutum* is unknown). Third stage larva identified as *H.* cf. *cornutum* has been reported as common in smelt (*Osmerus eperlanus*) from the German North Sea coast (Obiekezie et al., 1992), and there is also a rare finding of adult and fourth stage larval of *H. cornutum* in farmed cod *Gadus morhua* reared in northern Norway (MacKenzie et al., 2009). Here, the partial LSU, *cox*2 and ITS sequences of *H. cornutum* from its type host, the bluefin tuna, are reported for the first time. Phylogenetic analyses revealed a close genetic relationship between *H. cornutum* and other *Hysterothylacium* species, including *H. pelagicum* from *C. hippurus*, *Hysterothylacium australe* from the yellowtail amberjack *Seriola lalandi*, as well as *Hysterothylacium persicum* from the blue mackerel *Scomber australasicus*. These new molecular data may help clarifying the unresolved taxonomy of *Hysterothylacium* (Pantoja et al., 2016), the *H. cornutum* life cycle, and aid marine metabarcoding.

*Hysterothylacium aduncum* found free within the stomach lumen of one fish, is very common in marine fish in Norway (Berland, 1961). Adults and larvae of *H. aduncum* have previously been reported in bluefin tuna from Norway (Berland, 1961), but considering the amount of prey fish devoured by the tunas, this parasite seems unexpectedly rare. The presence of *Hysterothylacium* spp. in the gastrointestinal tract of bluefin tuna is a potential quality concern for the industry (Bao et al., 2021). This issue arises from the ability of these parasites to migrate from the tract and crawl out of the mouth, gills or vent to the body surfaces during cold storage or transportation. Notably, bluefin tuna anglers in western Norway have documented by photographs, the presence of over 40 large nematodes resembling adult *H. cornutum* in the stomach of one tuna captured in 2022 (M. Wiech, pers. obs.). To mitigate such quality concerns, prompt evisceration and thorough cleaning of the gills and pharyngeal cavities are recommended.

## 5 Conclusions

The present study documents, for the first time, the occurrence of *A. simplex* (s. s.) and *A. pegreffii* in large adult Atlantic bluefin tuna, revealing a previously undocumented host-parasite relationship and meriting attention regarding potential food safety concerns. The presence of crater-like ulcers in the stomach, associated with *A. simplex* (s. l.) larvae, suggests a pathological impact of these anisakids on the tuna host. This highlights the need to understand the pathogenic effects of anisakid infections on individual fitness and host populations in commercially important fish species. Furthermore, the detection of larvae on the caeca and intestines underscores the need for further investigation on tuna's muscle to disregard potential food safety concerns associated with raw tuna consumption. The molecular characterization of *H. cornutum* provides important data that contribute to resolve the taxonomy of the genus *Hysterothylacium*, in addition to aiding future studies on the parasite's life cycle.

## CRediT authorship contribution statement

**Miguel Bao:** Writing – review & editing, Writing – original draft, Visualization, Methodology, Investigation, Formal analysis, Conceptualization. **Arne Levsen:** Writing – review & editing. **Lucilla Giulietti:** Writing – review & editing, Project administration. **Martin Wiech:** Writing – review & editing, Resources, Conceptualization. **Keno Ferter:** Writing – review & editing, Resources. **Egil Karlsbakk:** Writing – review & editing, Investigation. **Paolo Cipriani:** Writing – review & editing, Investigation, Conceptualization.

## Declaration of generative AI and AI-assisted technologies in the writing process

During the preparation of this work, the author(s) used ChatGPT to improve the readability of the present manuscript. After using this tool/service, the author(s) reviewed and edited the content as needed and take(s) full responsibility for the content of the publication.

## Declaration of competing interest

The authors declare they have no competing interests.
